# Houseflies harbor less diverse microbiota under laboratory conditions but maintain a consistent set of host-associated bacteria

**DOI:** 10.1038/s41598-022-15186-7

**Published:** 2022-07-01

**Authors:** Anna Voulgari-Kokota, Leo W. Beukeboom, Bregje Wertheim, Joana Falcao Salles

**Affiliations:** Groningen Institute for Evolutionary Life Sciences (GELIFES), University of Groningen, P.O. Box 11103, 9700 CC Groningen, The Netherlands

**Keywords:** Ecology, Microbiology, Molecular biology

## Abstract

The housefly (*Musca domestica*) is a wide-ranging insect, often associated with decaying matter from livestock and humans. The septic environments in which houseflies live are believed to be a rich source for microbial acquisition. Although the housefly can harbor a wide range of microorganisms, it is not yet well known which microbes are always recurrent, which are dispensable and which environmentally dependent. In the present study, we aim at identifying which microbes are recurrently associated with the housefly gut throughout the species’ life cycle and whether their acquisition relies on the fly’s living environment. We surveyed three housefly strains—two of them kept under standard laboratory conditions for a long time and one wild-caught. To track any shifts happening throughout the lifecycle of the housefly and to test the consistency of the revealed microbial communities, we sampled houseflies at five developmental stages over the course of four consecutive generations. Both the bacterial and fungal microbiota of five developmental stages were studied for all samples, using amplicon sequencing for the 16S and ITS1 rRNA gene, respectively. Results revealed diverse microbial communities yet consistent for each of the two distinct sampling environments. The wild-caught population showed a more diverse and more distinct gut microbiota than the two laboratory strains, even though the strain was phylogenetically similar and shared geographic origin with one of them. Two bacterial genera, *Myroides* and *Providencia*, and two yeasts, *Trichosporon* and *Candida tropicalis,* were present in all sampled larvae and pupae, regardless of the strain. Analysis of the provided diet revealed that the flies acquired the yeasts through feeding. Our main findings show that houseflies might lose microbial diversity when reared in controlled environments, however they can maintain a consistent set of bacteria. We conclude that although the environment can facilitate certain microbial transmission routes for the housefly, and despite the fungal microbiota being largely acquired through diet, the larval bacterial gut microbiome remains relatively consistent within the same developmental stage.

## Introduction

The common housefly, *Musca domestica* is a widespread insect, commonly found thriving on organic leftovers of livestock and humans that are rich in microbes^1^. Most early studies on housefly–associated microorganisms focused on the fly’s capacity to vector pathogens^2–7^. Although the need to investigate potential public health risks related to the housefly remains actual^8^, the study on the beneficial effect of microbes on host health has become increasingly relevant. This is because *M. domestica* is considered suitable for mini-livestock farming, combining organic waste bio-remediation and commercial feed production^9,10^. This requires thorough knowledge of the insect’s microbiota both in terms of potential pathogens and of beneficial microbes which contribute to development.

It is well known that animal physiology is affected by interactions with host-associated microbial communities. These interactions are associated with a range of host phenotypes^11^ and are linked to essential functions such as digestion, nutrition, and immunity^12^. In insects, their relatively short life cycle allows studying host-associated microbiome shifts occurring in response to development, biotic interactions, abiotic conditions, or diet. For instance, early studies have pointed out the significance of bacteria for housefly development^13,14^, as larvae failed to grow in sterile environments. Later studies indicated the importance of specific bacterial taxa, such as *Providencia rettgeri*, which promoted normal development^15–17^, or Enterobacteriaceae, which were symbiotic^18^. Moreover, community analyses have shown that the complexity and composition of the housefly bacterial microbiota vary between developmental stages^19,20^, habitats^21–23^, and geographic locations^24^. Not all of the aforementioned studies, however, address the potential role of other components of the housefly microbiome, such as fungi, while even less is known about the stability of the housefly microbiome over developmental stages and multiple generations, as well about the microbes which are acquired by the immediate substrate where housefly larvae grow in.

In the present study, we investigated the potential influence of population background, geographic origin, and sampling environment on the internal microbial communities of *M*. *domestica*. Moreover, we aimed to determine whether there is a core set of microbial phylotypes present in the house fly gut microbiota, regardless of the population origin. For that, we sampled three housefly populations over four consecutive generations. Two of them were collected in the same cattle farm in the Netherlands, of which one had subsequently been reared in the laboratory for three years (> 50 generations), and one was wild-caught. The third fly strain originated from Spain and had also been kept in the laboratory for over 100 generations. We sampled eggs, larvae, pupae, newly emerged adults, and three day-old adults from each of these populations and we characterized the associated bacterial and fungal communities, using a fraction of the 16S ribosomal RNA and the internal transcribed spacer 1 (ITS1) region, respectively.

We hypothesized that host genetic background and sampling location will influence the microbiome composition across all developmental stages, but that prolonged laboratory maintenance will reduce microbial diversity. We aimed at revealing which microorganisms were recurrently present in the housefly gut, regardless of strain and origin, and determine their possible acquisition routes. We also predicted distinctive microbial shifts across the housefly’s lifecycle, associated with the various developmental stages. Finally, we did not expect strong fluctuations over generations, with the exception of the wild-caught strain that was taken into laboratory culture.

## Materials and methods

### Housefly strains and culturing

Adult house flies (n =  ~ 600) were sampled from a cattle farm at Gerkesklooster (GK) in the Netherlands in June 2020, and were transferred to the laboratory for further handling. A subset of flies was stored at − 20 °C immediately (~ 50), and the remainder was used to start a laboratory culture. We refer to this wild-caught line as GK0 (for generation 0). We also used a housefly strain, that originated from the same sampling site but had already been established three years earlier, and reared in the laboratory for over 50 generations (GK50), and a housefly strain that was initially collected in Barcelona, Spain in 2015, and has been reared in the same laboratory for over 100 generations (SP100). Flies were kept in identical cages, and the number of adults in each cage was exactly 100, with a sex ratio of 1:1. All adults were provided with sterile water, sterile 20% sucrose solution and milk-powder ad libitum. Adult flies were provided with cups containing egg-laying substrate. The substrate was composed of wheat bran (75%), flour (12%), milk powder (9%), and inactivated yeast (4%). All cages were kept at 25 °C with a 12 h light/12 h dark cycle.

### Sample collection

We sampled eggs, larvae, pupae, newly emerged adults and three-day-old adults for all three housefly strains over four consecutive generations (Supplementary File 1). For egg collection, egg-laying cups were removed from each cage after four hours to ensure that all eggs were laid around the same time. The eggs of each strain were counted and mixed, a sample (50 mg) was taken for every strain, and then the remaining eggs from each strain were placed on additional substrate keeping the same egg/substrate ratio (4 eggs/1 g of the substrate) at all times. Three days after the housefly eggs hatched into larvae, one sample with four larvae was taken from each container. Three days later, we sampled four pupae from each container. Subsequently, we sampled four newly emerged adults from each container as soon as they emerged from their cocoons. After moving the new generation of adults from each container into cages, we finally sampled three-day-old adults again, before we provided them with the egg-laying substrate so they would produce the next generation. All samples from larvae, pupae and adults were collected in triplicates, using sterile tweezers, and all samples were washed with sterile PBS/EDTA solution immediately after sampling. Dissection of the guts of larvae, pupae, and adults was conducted under sterile conditions, and along with whole intact eggs, all samples were stored at -20 °C. Additionally, we sampled the fresh egg-laying substrate which was provided to each generation of flies (four samples in total), to investigate its bacterial and fungal microbiota.

### Phylogenetic relationships of the sampled housefly strains

Twelve individuals from each of the three housefly strains were picked randomly and total genomic DNA was extracted following the high salt protocol^25^. The mitochondrial cytochrome C oxidase subunit I (COI) gene sequence for each of the sampled individuals was amplified with the following PCR conditions: initial denaturation at 95 °C (2′); 30 cycles of DNA denaturation at 94 °C (30″), primer hybridization at 50 °C (30″), strand elongation at 72 °C (45″); and a final elongation at 72 °C (7′). The PCR products were purified using ExoSAP-IT™ PCR Product Cleanup Reagent (Thermo Fisher Scientific, Vilnius, Lithuania). Both for the PCR amplification and the Sanger sequencing, the following primers were used: forward LCO-1490 (5′-GGTCAACAAATCATAAAGATATTG-3′), reverse HC02198 (5′TAAACTTCAGGGTGACCAAAAAATCA3′)^26^. The amplicons were aligned and a phylogeny was constructed to examine the phylogenetic relationship between all three fly strains (Supplementary File 2). Details of the phylogenetic analysis and the constructed phylogenetic tree based on the revealed phylotypes are described in Supplementary File 2.

### Next generation sequencing for microbial metabarcoding

Total genomic DNA was isolated from all specimens sampled for microbial metabarcoding, using the Qiagen PowerSoil DNA extraction kit, following the manufacturer’s instructions, with an additional lysis step to ensure the inclusion of hard-to-lyse microbes. Quality control was conducted with qPCR to detect the gene copy numbers in each sample for the 16S rRNA and the ITS1 rRNA. The extracted DNA was then used to construct two libraries for the bacterial 16S rRNA and the fungal ITS1 gene, with dual indexing. The overall library preparation included purification, normalization based on the qPCR quality control, and pooling. Sequencing consisted of a 2 × 300 bp MiSeq run (Illumina) on the V4 region of the bacterial 16S rRNA (515F–926R primer pair) and a run on the fungal ITS1 (ITS1F*_Nextera - ITS2*Nextera primer pair)^27^. Library preparation and sequencing were performed at the Genomics Center of the University of Minnesota.

### Data analysis

Quality filtering of the raw data, denoising and representative sequence picking was conducted with DADA2^28^ using default parameters on the QIIME2 v 2020.6 platform^29^. Representative amplicon sequence variants (ASVs) were aligned using MAFFT^30^ and an unrooted phylogenetic tree was constructed with Fasttree^31^. Taxonomic assignments for the bacterial dataset were conducted using the SILVA 138 reference database^32^, whereas taxonomic assignments for the fungal dataset were performed using the UNITE reference database^33^. The confidence level of all assignments was 0.97. Forward and reverse sequencing reads were submitted to the Sequence Read Archive (SRA) under BioProject ID PRJNA806649.

Data were further analyzed in R 4.0.3^34^. using the packages phyloseq v1.22.3^35^, microbiome^36^, ape^37^, codyn^38^ and vegan v2.5-2^39^, for diversity and community composition analysis. The bacterial dataset was filtered to exclude sequences annotated as chloroplasts or mitochondria. All samples were checked to confirm that they had more than 1,000 remaining reads after filtering. The only samples which were omitted from further analysis where the ones with fewer than 1000 acquired reads.

Alpha-diversity of samples was calculated using the absolute richness, Shannon index, and Phylogenetic Diversity (PD)^40^. Beta diversity was visualized with nonmetric multidimensional scaling (NMDS) ordination based on weighted UniFRAC distance matrices, considering both microbial phylogenetic relations and relative abundances of detected phylotypes. Comparison of alpha diversity between sample groups was done with ANOVA tests for Shannon values and general linear models for all indices. Beta dispersion was calculated as a metric for beta diversity within sample groups by calculating the average distance of group members to the group centroid in the multivariate space generated by a distance matrix (in our case a UniFRAC distance matrix)^41^. PERMANOVA/Adonis^42^ was used to test microbial composition homogeneity between group levels by setting various factors from the sample metadata as discriminative factors. Bacterial and fungal distance matrices were correlated with Mantel tests, based on Pearson’s product correlation^43^. Finally, random forest analysis was used to assign bacterial and fungal microbial communities to different groups and estimate the factors which give the lowest errors (out-of-basket errors) for correct classification with the packages varSelRF v0.7-8^44^ and randomForest v4.6-14^45^. Aggregated predictions were made to classify samples according to sampling environment (farm/lab) or country of origin (Netherlands/Spain), using *N*_*tree*_ = 10,000. All ordination and diversity graphs were constructed with ggplot2 v3.0.0^46^.

## Results

The copy numbers for 16S and ITS1 rRNA, and the sequencing depth for all samples are presented in Supplementary File 3 (qPCR data, Sequencing Rarefaction Curves). An average of 14,265.25 reads per housefly sample for the V4 16SrRNA and 16,149.4 reads per housefly sample for the ITS1 were retained after quality filtering. After quality filtering of the egg-laying substrate samples, an average of 10,371.75 reads were retained per sample for the V4 16SrRNA, and an average of 25,479.75 reads were retained per sample for the ITS1 region. The extracted DNA from newly emerged adult houseflies of the Spanish laboratory strain (12 samples in total, newly emerged adults, three replicates from four generations, strain SP100) returned a low copy number for the fungal ITS1 (qPCR data, Supplementary File 3) and a low number of acquired sequencing reads; they were therefore omitted from any further analysis of the fungal microbiota. In addition, the mitochondrial COI phylogeny showed that the Dutch wild-caught strain and the Dutch laboratory strain, which were sampled from the same locality at different times, are in close proximity and form a separate clade from the Spanish lab strain phylotypes (Supplementary File 2).

### The housefly microbiota alpha-diversity is determined by sampling environment

**Figure 1:**
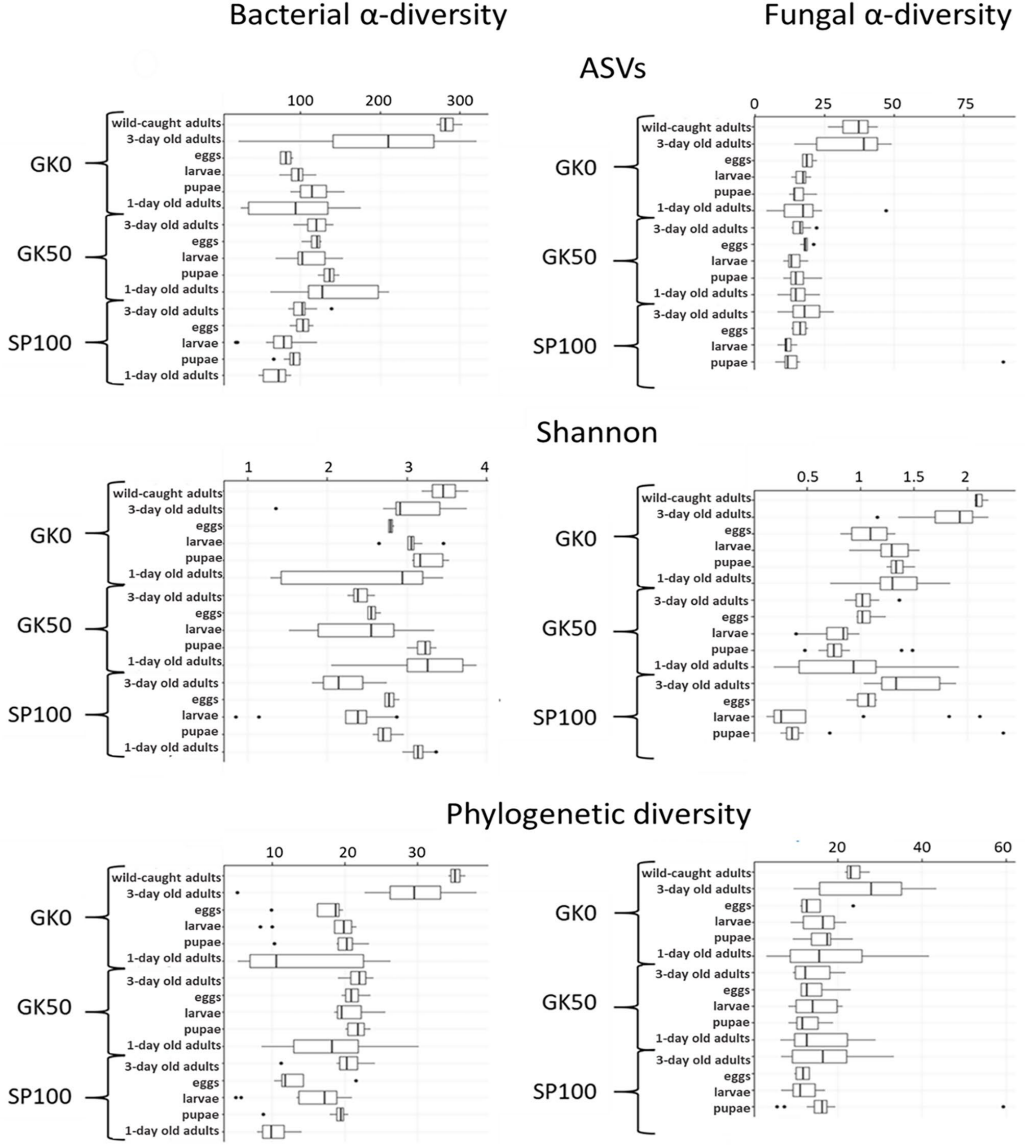
Bacterial and fungal alpha diversity of the microbial communities from developmental stages of three *Musca domestica* strains, estimated with three diversity metrics: the number of Amplicon Sequence Variants, Shannon index, and Faith’s Phylogenetic Diversity. The line in each boxplot represents the median of all values, with the upper limit of the box being the first and the lower limit of the box being the third quartile. Each boxplot represents all samples of one housefly strain (GK0, GK50, or SP100) per developmental stage (3 day old adults, eggs, larvae, pupae, 1 day old adults), i.e. three biological replicates for four generations. For the GK0 strain, one separate boxplot represents the wild-caught adults immediately after collection (adults from the farm). GK0: field population, the Netherlands; GK50: laboratory population originally collected in the Netherlands; SP100: laboratory population originally collected in Spain.

Absolute richness (number of ASVs), Shannon index, and Phylogenetic diversity for all housefly strains and developmental stages are shown in Fig. 1. The highest bacterial alpha diversity was observed for the wild-caught housefly population GK0. Strain was an important factor for separating Shannon biodiversity levels both for newly emerged (F = 4.37, *P* < 0.05) and 3-day-old adults (F = 14.70, *P* < 0.001), as shown by single-factor ANOVA. Pairwise comparisons between strains showed that GK0 was significantly different from the Dutch laboratory strain GK50 (newly emerged adults: F = 4.83, *P* < 0.05, 3-day-old adults: F = 12.64, *P* < 0.01) and the Spanish laboratory strain SP100 (newly emerged adults: F = 5.79, *P* < 0.05, 3-day-old adults: F = 18.49, *P* < 0.001), in terms of bacterial alpha diversity. However, the adults of the two laboratory strains (GK50 and SP100) showed no significant difference (*P* > 0.05). Comparisons for fungal alpha diversity between all strains found similar results with the respective comparisons for the bacterial alpha diversity for the newly emerged adults (F = 4.55, *P* < 0.05) and the 3-day-old adults (F = 29.11, *P* < 0.001). The GK0 strain harbored a more diverse fungal community than the GK50 strain (F = 6.43, *P* < 0.05 and F = 65.00, *P* < 0.001, for the newly emerged and the 3-day-old adults, respectively) and the Spanish laboratory strain SP100 (F = 12.23, *P* < 0.05, for the 3-day-old adults).

In general, adult age was a significant factor when comparing the bacterial alpha diversity of newly emerged and 3-day old adults within the Dutch laboratory strain (F = 19.90*,* *P* < 0.001) and the Spanish laboratory strain (F = 97.18, *P* < 0.001), with newly emerged adults showing more diverse communities. On the contrary, bacterial alpha diversity did not differ between newly emerged and 3-day old adults for the wild-caught strain. An opposite pattern was found for the fungal alpha diversity, where only the wild-caught strain (GK0) showed a significant difference between 1-day old and 3-day old adults (F = 16.00, *P* < 0.001).

Similar to the housefly adults, the larvae from the GK0 strain harbored more diverse bacterial communities, with the housefly strain being an important factor for the overall comparison of all larvae (F = 8.70, *P* < 0.001). The larval bacterial alpha diversity from this strain was significantly higher than GK50 and SP100 (F = 11.02, *P* < 0.01 and F = 20.26, *P* < 0.001, respectively). In contrast, the two laboratory strains did not differ from each other (*P* > 0.05). The same pattern was found for the fungal alpha diversity for larvae (all larvae: F = 9.22, *P* < 0.001, GK0 with GK50: F = 49.20, *P* < 0.001, GK0 with SP100: F = 12.18, *P* < 0.05 and GK50 with SP100: *P* > 0.05) and pupae (all pupae: F = 13.98, *P* < 0.001, GK0 with GK50: F = 32.24, *P* < 0.001, GK0 with SP100: F = 22.99, *P* < 0.001 and GK50 with SP100: *P* > 0.05). However, we did not find similar results for the bacterial diversity of the pupae, for which the Dutch wild and the laboratory strains showed similar levels (*P* > 0.05).

### Bacterial but not fungal alpha diversity is reduced after introduction into the laboratory

**Figure 2:**
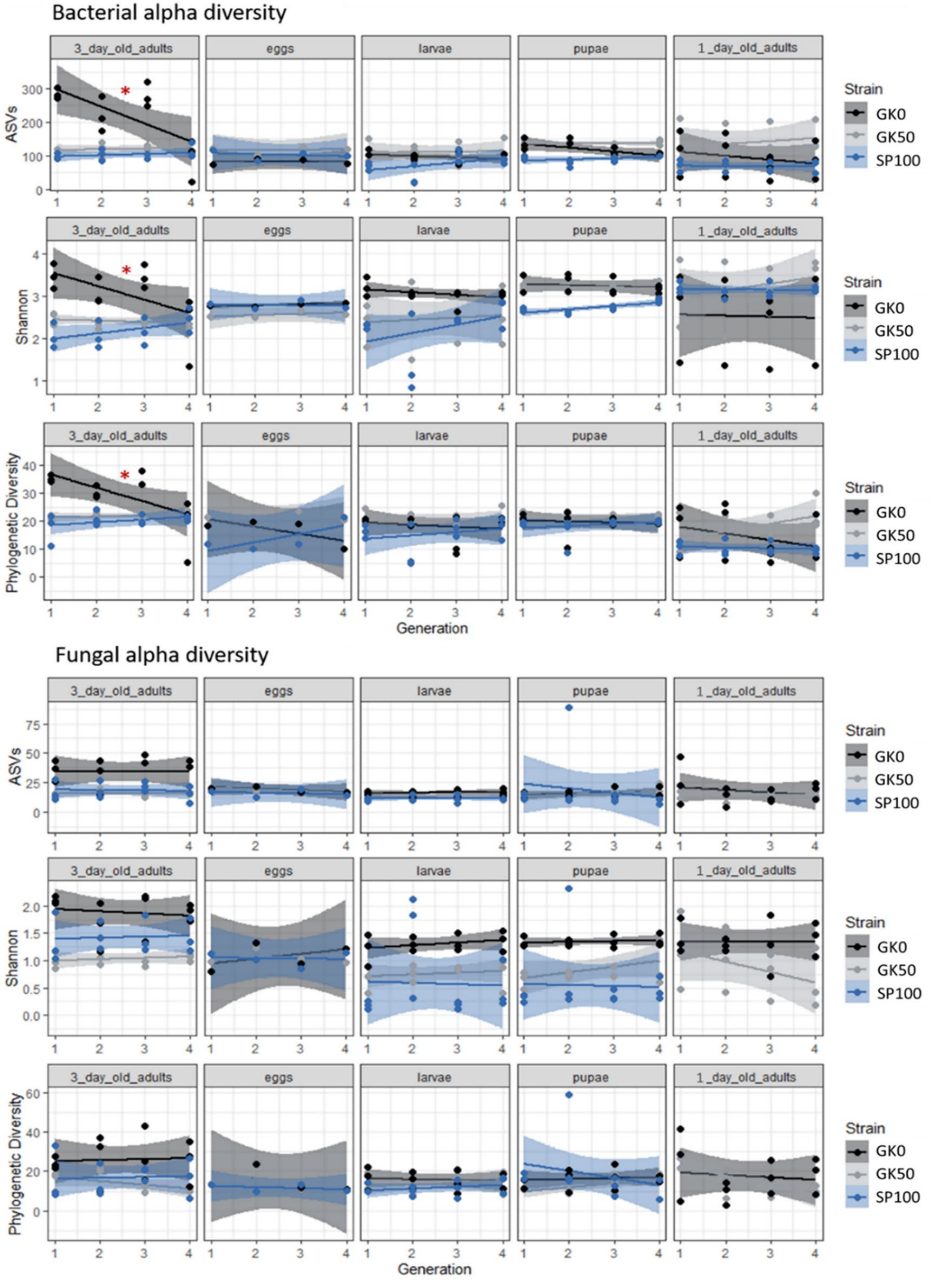
Bacterial and fungal alpha diversity from developmental stages of three *Musca domestica* strains over four generations, estimated with three diversity metrics: richness represented by the number of different Amplicon Sequence Variants, Shannon index, and Faith’s Phylogenetic Diversity. Each subplot represents a combination of one housefly strain (GK0, GK50, or SP100) and one developmental stage (3 day-old adults, eggs, larvae, pupae, 1-day old adults). Lines show the linear regression for each housefly strain alpha diversity values across generations. The shaded field around the lines stands for the standard error of the linear regression. Alpha diversity decreased significantly only for the bacterial communities derived from older adults of the GK0 strain, as the red asterisk suggests.

Bacterial and fungal alpha diversities were estimated separately for samples from each of the four generations (Fig. 2). For the GK0 strain, the absolute numbers of ASVs, Shannon Diversity, and Phylogenetic Diversity decreased for the bacterial communities derived from older adults throughout the four sampled generations (Fig. 2). The first generation of the strain consisted of the adults caught in the cattle farm, whereas the following generations were reared under laboratory conditions. Linear model testing for all three metrics returned significant results for this sample subset (ASVs: R^2^ = 0.46, *P* < 0.05; Shannon: R^2^ = 0.32, *P* < 0.05; Phylogenetic diversity: R^2^ = 0.39, *P* < 0.05), indicating a decline in alpha diversity over successive generations. No other combination of strain and developmental stage returned statistically significant results for the three alpha diversity metrics, indicating alpha diversity remained similar across generations. For the fungal communities, fungal alpha diversity did not decrease with the transfer of the GK0 adults into the laboratory; it remained similar for all strains and life stages across the four generations.

### Beta diversity of housefly associated microbial communities is more similar between laboratory strains

**Figure 3:**
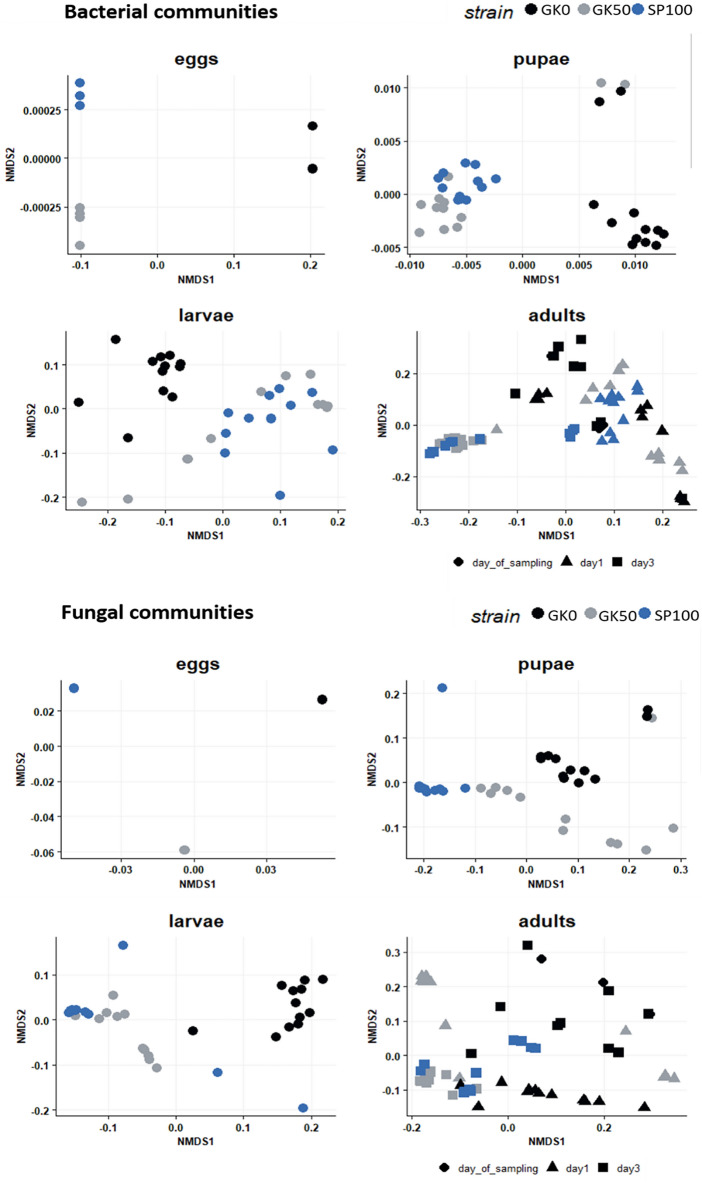
(**a**) Bacterial communities for each developmental stage of three housefly strains based on weighted Unifrac. NMDS stresses: 9.05e-05 (eggs), 0.09 (larvae), 0.08 (adults),0.06 (pupae) (**b**) Fungal communities for each developmental stage based on weighted Unifrac. NMDS stresses: 9.91e-05 (eggs), 0.07 (larvae), 0.06 (pupae), 0.15 (adults).

Whether the housefly strain had been kept in the laboratory or was wild-caught served as a more significant explanatory factor for the shaping of the bacterial and fungal microbiota composition than the country of origin (Netherlands/Spain), for all developmental stages (Permutational multivariate analysis of variance, Table 1). Permutation test for homogeneity of dispersions showed that the bacterial communities from all developmental stages had similar beta dispersion values (average distance to the group centroid) for each of the three housefly strains (*P* > 0.05). The results for the fungal communities were similar, with the exception of pupae. More specifically, the Spanish laboratory strain showed a significantly lower beta-dispersion than the respective Dutch laboratory strain (*P* < 0.01**), thus lowering the significance of the respective PERMAVONA result (Table 1). To further investigate these factors potentially explaining the observed microbial beta diversity, we tested them as predictors for assigning samples to groups. Again, the sampling environment was a more accurate factor in predicting community structure for bacterial and fungal microbiota, since the assignment errors were lower than those for assignment according to the country of origin. Especially for the fungal communities, both larvae and adults were more accurately assigned to the correct sampling environment than to the correct country of origin (Random forest classification, Table 2). Furthermore, for eggs, larvae, and pupae, the two laboratory strains were similar, whereas the wild-caught farm strain was distinct from the remainder of the samples (NMDS ordination, Fig. 3). Adults from the two laboratory strains were also more similar; however, the clustering patterns were less clear. Indeed, only a small proportion of the total adult microbiota variance could be explained by rearing history in the laboratory (Table 1).

**Table 1:** Permutational multivariate analysis of variance for bacterial and fungal communities associated with each housefly developmental stage, based on weighted UniFrac distance matrices.

Factor tested	Sampling environment of Dutch strains (farm/laboratory)			Geographic origin of laboratory strains (Netherlands/Spain)			
Housefly strain	GK0	R^2^	*P*	GK50	R^2^	*P*	SP100
**Bacterial communities**							
Developmental stage	Eggs	0.99374	0.038*	Eggs	0.77592	0.028*	Eggs
	Larvae	0.27373	0.002**	Larvae	0.03116	0.468	Larvae
	Pupae	0.48161	0.001***	Pupae	0.20533	0.001***	Pupae
	1-day old adults	0.08774	0.009**	1-day old adults	0.04415	0.124	1-day old adults
	3-day-old adults	0.07982	0.012*	3-day-old adults	0.04154	0.13	3-day-old adults
**Fungal communities**							
Developmental stage	Eggs	0.94606	0.031*	Eggs	0.97014	0.033*	Eggs
	Larvae	0.73062	0.001***	Larvae	0.08214	0.115	Larvae
	Pupae	0.58297	0.006**	Pupae	0.53598	0.001***	Pupae
	1-day old adults	0.16716	0.001***	1-day old adults			1-day old adults
	3-day-old adults	0.16805	0.001***	3-day-old adults	0.09626	0.005**	3-day-old adults

Beta-diversity comparisons are conducted between the wild and laboratory Dutch strains, and between the Dutch and Spanish laboratory strains.

**Table 2:** Out of basket errors (%) after assigning samples to two categories according to sampling environment (farm/lab) or country of origin (Netherlands/Spain) with random forest classification.

Developmental stage	Bacterial communities		Fungal communities	
	Sampling environment (n = 2)	Country of origin (n = 2)	Sampling environment (n = 2)	Country of origin (n = 2)
Eggs	0	0	0	0
Larvae	0	0	2.78	11.11
Pupae	5.56	0	2.78	2.78
Adults	0	6.94	5.56	6.97

**Figure 4:**
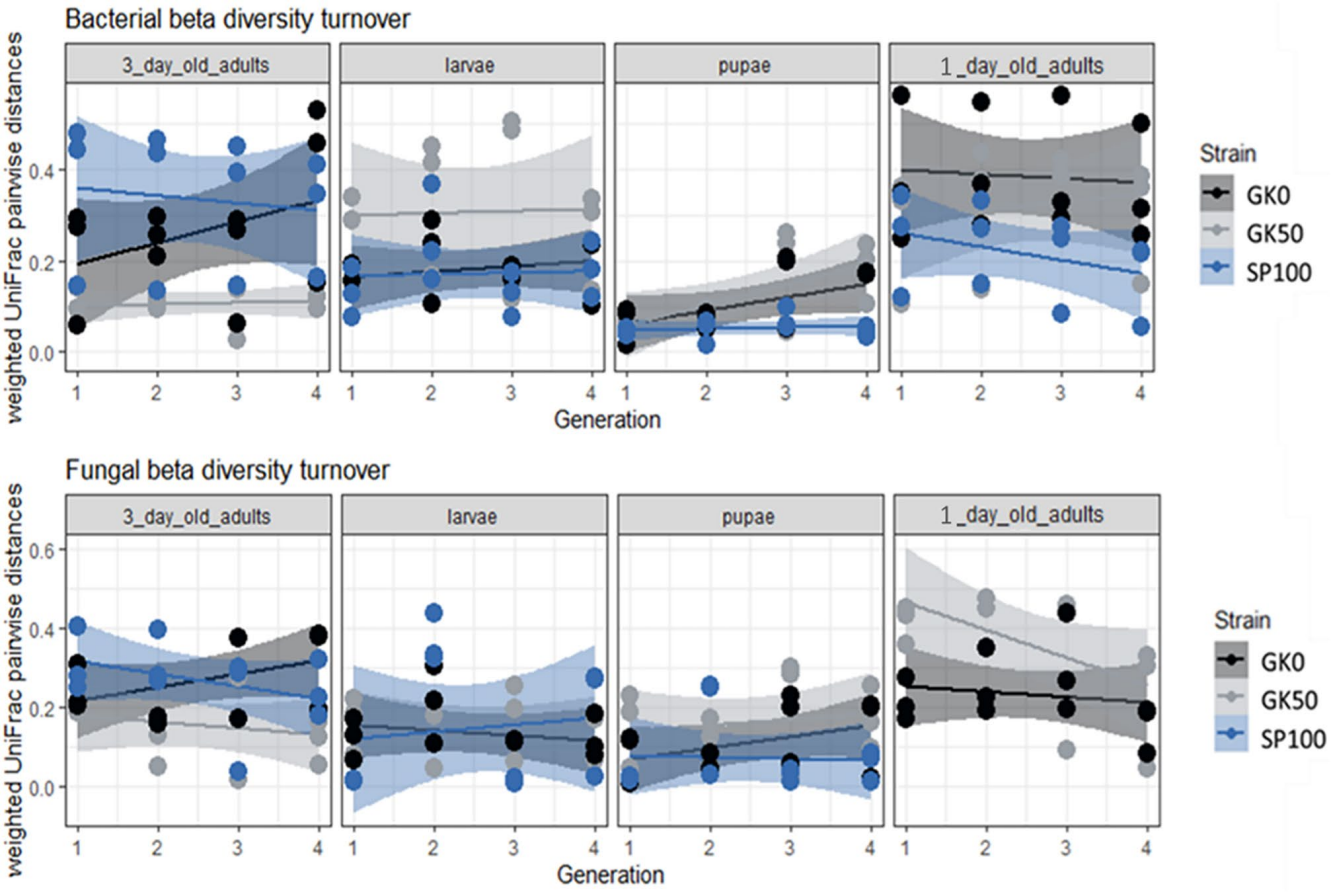
Weighted UniFrac pairwise distances for all housefly developmental stages of three strains over four consecutive generations as a metric for beta diversity turnover for bacterial and fungal communities. Beta-diversity patterns showed no significant shifts between housefly generations. Lines show the linear regression for each housefly strain beta diversity values across generations. The shaded field around the lines stands for the standard error of the linear regression. Beta diversity did not shift significantly across generations for any housefly strain.

To determine whether the culturing of the strains in the laboratory or the transfer from field to laboratory lead to substantial community shifts across generations, we evaluated the microbial turnover over generations, for each strain and stage (Fig. 4). Beta-diversity patterns showed no significant shifts between housefly generations for the GK0 strain, similarly to the other two laboratory strains.

Beta diversity analysis of the housefly-associated microbial communities showed that the fungal communities were less consistent than the bacterial communities within the same developmental stage, with the revealed bacterial communities being more homogeneous within the same stage. More specifically, the beta dispersion value (average distance to the group centroid) for the bacterial communities retrieved from housefly eggs was 0.20 compared to 0.41 for fungal communities. Microbial communities of larvae and pupae were also more homogeneous in bacterial composition (beta dispersion: 0.15 and 0.10, respectively) than in terms of fungal composition (beta dispersion: 0.29 and 0.22, respectively). Finally, fungal communities varied more in the case of newly emerged adults (beta dispersion: 0.40) and 3-day old adults (beta dispersion: 0.36) than bacterial communities from newly emerged adults (beta dispersion: 0.19) and 3-day old adults (beta dispersion: 0.20).

Upon correlating the distance matrices of both the bacterial and the fungal dataset, we detected a weak co-variance of the bacterial and fungal communities within the whole dataset (r = 0.27, *P* < 0.001). To investigate whether this significant correlation was an effect of strain or life cycle stage specificity, we ran the same tests within subsets of all samples. We found statistically significant correlations, both when we investigated each strain and each stage of the housefly life cycle, separately (Table 3). When we conducted the same tests within each developmental stage of the same housefly strain, we detected significant correlations for all subsets except for the microbial communities associated with eggs from all strains and pupae from the SP100 strain (Table 3).

**Table 3:** Mantel correlations of *M. domestica* bacterial and fungal microbial communities, based on weighted UniFrac distance matrices.

	Mantel statistic (r)	*P*-value
**Whole dataset**	0.27	< 0.001***
Eggs (n = 12)	0.68	< 0.01**
Larvae (n = 36)	0.42	< 0.001***
Pupae (n = 36)	0.49	< 0.001***
Adults (n = 72)	0.31	< 0.001***
**Housefly strain**		
GK0 (n = 52)	0.30	< 0.001***
GK50 (n = 52)	0.33	< 0.001***
SP100 (n = 52)	0.71	< 0.001***
**Housefly strain × developmental stage**		
GK50 eggs (n = 4)	0.05	> 0.05
GK50 larvae (n = 12)	0.60	< 0.01**
GK50 pupae (n = 12)	0.63	< 0.001***
GK50 adults (n = 24)	0.69	< 0.001***
GK0 eggs (n = 4)	0.25	> 0.05
GK0 larvae (n = 12)	0.68	< 0.001***
GK0 pupae (n = 12)	0.89	< 0.001***
GK0 adults (n = 24)	0.24	< 0.01**
SP100 eggs (n = 4)	0.03	> 0.05
SP100 larvae (n = 12)	0.44	< 0.05*
SP100 pupae (n = 12)	− 0.11	> 0.05
SP100 adults (n = 24)	0.49	< 0.001***

### Microbial composition of the laboratory strains is simpler, and a set of recurring microbial phylotypes can be distinguished

**Figure 5:**
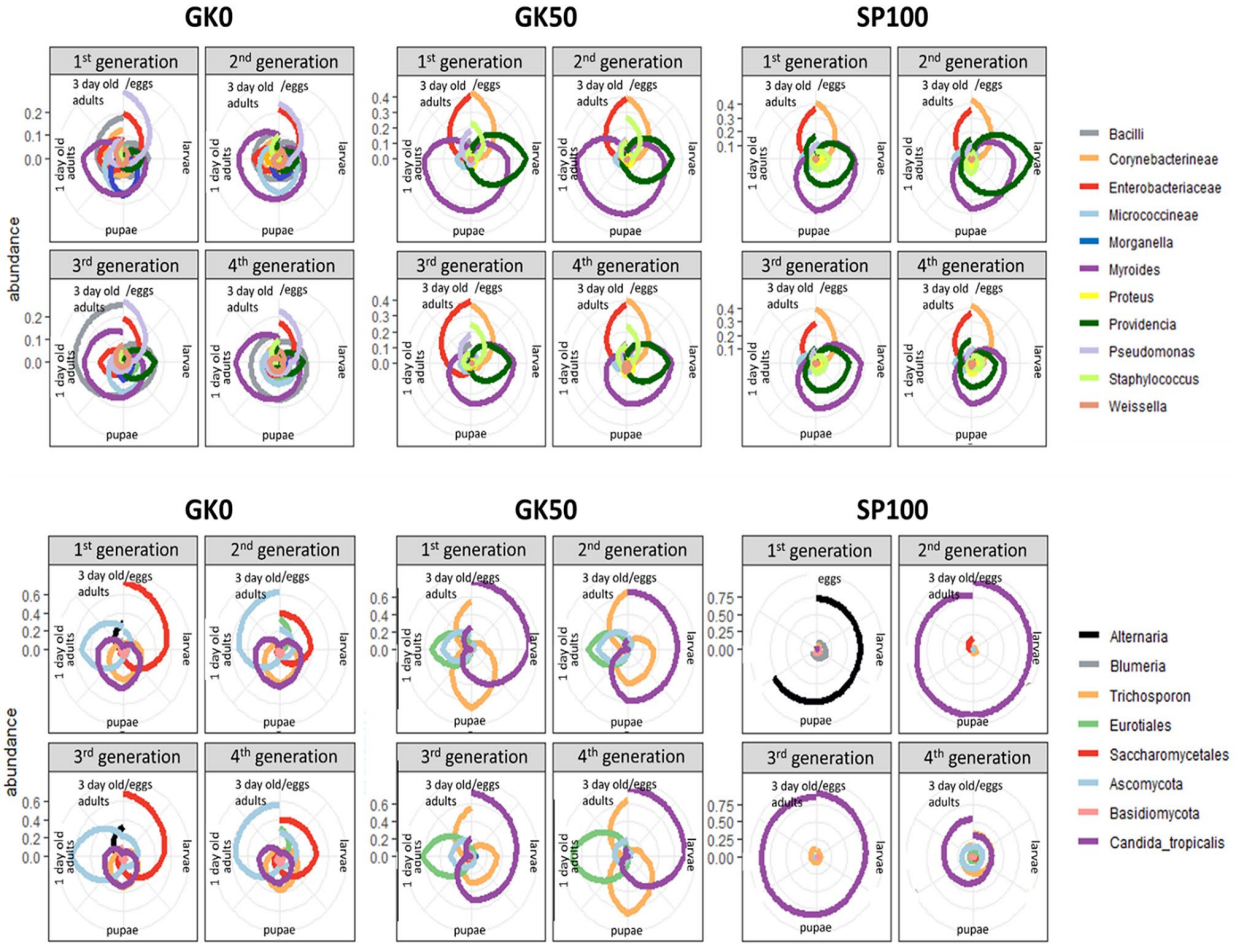
Microbial composition of developmental stages of three housefly strains over four generations. Each separate plot stands for all samples (three replicates for every developmental stage) of the same housefly strain and the same generation. Each circle represents the housefly life cycle from the egg stage to the adult stage. Lines stand for the relative abundance of bacterial (top) or fungal (down) taxa. Bacterial and fungal taxa are included if the relative abundance of the phylotypes assigned to them are detected at a percentage of at least 5% in the whole dataset.

Two bacterial genera, *Providencia* and *Myroides*, were detected in all samples from larvae and pupae of all housefly strains and generations (Fig. 5). These same taxa were not detected in the samples from the provided substrate (Microbial composition, Supplementary File 3). Furthermore, the genus *Morganella* was detected mainly from samples of the wild strain (GK0) larvae and pupae. In contrast, the genus *Proteus* was detected in larvae and pupae of the laboratory strains (Fig. 5). Again, none of these bacterial taxa were found in the fresh substrate we provided to the flies. Finally, the bacterial family of Enterobacteriaceae was more dominant in the adult fly guts of the two laboratory housefly strains (Fig. 5), along with the genera *Pseudomonas* and *Acinetobacter*. Both laboratory strains showed similar patterns of bacterial composition succession during the housefly life cycle, across the four generations. In contrast, the GK0 strain showed a more complex microbiota structure in line with its revealed higher alpha-diversity.

Two yeasts assigned to the fungal taxa *Trichosporon sp*. and *Candida tropicalis,* were prevalent in all larvae and pupae of our dataset (Fig. 5). The same taxa were detected in all four batches of the feed, which was provided as the egg-laying substrate (Microbial composition, Supplementary File 3), indicating that the flies acquire them from their environment. Sequences assigned to the fungal order Saccharomycetales were detected in all batches of the provided substrate as well. However, it was mainly found in flies from the wild-caught strain and was not dominant in flies from the laboratory strains (Microbial composition, Supplementary File 3).

## Discussion

Insect microbiota is an essential element of insect health, playing a pivotal role in organismal function and development. Microbial transmission is mainly facilitated through environmental pathways or active transfer from parents to offspring. In the case of houseflies, their habitats are typically characterized by rich microbial communities. Moreover, especially during their early development, they thrive on decomposing organic material, a rich source of various microorganisms. The main pathways through which houseflies acquire their associated microbiota include their habitat, food, and interactions with conspecifics.

We investigated the bacterial and fungal microbial communities of three housefly strains from two geographic origins. Two of the three strains had been reared in the laboratory for many generations, and one of them was collected from a cattle farm from which one of the laboratory strains also originated. This allowed us to examine the influence of the geographic origin—by comparing laboratory strains—and that of the sampling environment (laboratory or farm) on shaping the fly microbiota. All strains were reared under identical conditions, and samples were taken at five developmental stages during the life cycle over four consecutive generations. To our knowledge, this is the first housefly microbiota survey that simultaneously examines geographic origin, sampling environment and developmental stage over multiple generations with regard to the associated bacterial and fungal communities.

### The wild population harbors a more diverse microbial community than the laboratory strains

The adults directly sampled from the cattle farm showed the highest bacterial biodiversity among all adults. Furthermore, although there was a reduction in bacterial alpha diversity with the transfer of the wild-caught adults into the controlled laboratory environment, there was no such pattern of biodiversity reduction for larvae and pupae of the wild population during the rearing of the strain over four generations. Nevertheless, the wild population showed higher bacterial and fungal diversity for larvae, pupae, and adults compared with the laboratory strains. The rearing history of the strains in the lab seems to be the main factor influencing biodiversity, as the strain sampled from the cattle farm harbors a more diverse microbiota than those reared under laboratory conditions for multiple generations. This coincides with other microbial surveys of houseflies, which showed that flies from farms were associated with different bacterial communities depending on the habitat^23^ and with more diverse microbial communities when compared with houseflies sampled from hospitals in the same geographic region^24^. Furthermore, the age of the adults was connected with different bacterial biodiversity levels for the laboratory strains but not for the wild population. More specifically, newly emerged adults harbored richer bacterial communities than the three-day-old adults, both for the Dutch and the Spanish laboratory strains.

### Sampling environment is the main factor shaping the housefly microbiota

The sampling environment heavily influenced the microbiota structure of all housefly developmental stages. Most remarkably, eggs, larvae, and pupae from the two laboratory strains were much more similar in comparison to the wild strain, even though the environment and the provided diet were identical for all strains. Furthermore, the common geographic origin and the phylogenetic proximity of the Dutch wild strain and the Dutch laboratory strain did not seem to be of great importance for the microbiota structure. On the other hand, although harboring a more consistent microbial community within the same strain, adult flies were not separated that prominently according to housefly strain. To explain this pattern, we must consider the lifestyle of each developmental stage^20^. Eggs were placed on the same substrate to hatch and, consequently, the larvae of each housefly strain grew in the same substrate. At this stage, their immediate environment and their sole feeding source are the provided substrate, while the aggregations formed by larvae could mediate the social exchange of microbes. Adult flies, however, and especially older ones, live in a less defined environment. Although they were provided with the same feed, they are more mobile and do not live in constant and direct contact with each other. Therefore, it might be easier for larvae to obtain similar microbial communities within the same population.

When we compared the structure of the bacterial and the fungal communities from the samples of the whole dataset (all strains, stages, and generations), we found that even though the fungal microbiota was less diverse in terms of alpha-diversity within each sample, fungal communities were more diverse between samples. The less consistent profile of the fungal microbiota could mean that the acquisition of fungi is less controlled and more erratic compared to the acquisition of bacterial symbionts; these results confirm previous findings of housefly-associated bacteria and fungi^24^. Nevertheless, bacterial and fungal communities co-varied within all samples and both within and between housefly strains. Co-variation of the bacterial and the fungal microbiota has been reported in the case of many species^47^. A major gap in our knowledge is the extent to which this phenomenon can be attributed to selection by the host or to interactions within the microbiome. In our samples, larvae and adults show a co-variance of these two components of the microbiota within all housefly strains, pointing out that there might be interactions between bacteria and fungi. Interestingly so, the wild-caught strain adults show a weaker co-variance in comparison to the larvae, however whether this is an effect of the sampling environment cannot be answered here and demands further research.

### The housefly bacterial communities are more consistent than the fungal communities

The investigation of the bacterial community composition revealed several prevalent bacterial taxa reported as host-specific from previous studies. Two bacterial genera, *Providencia* and *Myroides*, were detected in all larvae and pupae regardless of the housefly strain and the generation. *Providencia* has been characterized as prevalent^16,17^ and promotes normal development of larvae^15^. *Myroides* sp. has also been found in houseflies in several bacterial microbiota surveys^16,48^; the functional role of the genus, however, remains obscure, although it has been hypothesized that it might promote wheat bran degradation and digestion^16^. The microbe was also detected in samples of wheat bran–based substrate, inhabited by housefly larvae, indicating horizontal transfer between the housefly larval gut and the larval substrate^21^. Furthermore, phylotypes assigned to the bacterial family of Enterobacteriaceae were more dominant in adult houseflies of all three strains. The family has been characterized as symbiotic for *M. domestica*, often found with enterococci in the housefly gut^18^.

Previous studies have also highlighted two other bacterial taxa as associated with *M. domestica*: *Morganella morganii*^15,21,23,49^ and *Proteus spp*.^18,21,23^. Both bacterial taxa are considered potentially pathogenic. *M. morganii* has been isolated from houseflies inhabiting wastelands^49^, and it has been reported as responsible for clinical infections^18^. Additionally, it caused mortality in laboratory-reared Mexican fruit fly larvae^50^. Both bacterial taxa have also been found in blowflies^22^, while their association with *M. domestica* is why the housefly is accepted as a putative vector for these microorganisms^18^. The presence demands deeper investigation on their association with the house fly and more profound testing on whether they can become dangerous for human health. In our dataset, the genus *Morganella* was mainly present in larvae and pupae of the wild strain, whereas *Proteus* was detected in larvae and pupae of the laboratory strains. At the same time, these taxa were not found in the substrate we provided to the flies, indicating acquisition through interaction between conspecifics. Interestingly, an early study by Greenberg and Klowden^4^ demonstrated that high levels of *Proteus mirabilis* in house fly larval guts suppressed the growth of two pathogenic microorganisms, *Salmonella typhimurium* and *Pseudomonas aeruginosa*. Therefore, the close association with the housefly gut might aid the fly health through niche occupation.

The presence of specific bacterial phylotypes in all housefly larvae included in the current study, regardless of the strain, and the relatively stable maintenance of larval gut bacteria over several generations for the GK0 strain imply that there is a vertical bacterial transmission route, independent of the feeding substrate provided to newly emerging larvae. This is an interesting finding, however little is known on the mechanisms which would facilitate such a pathway in the housefly. One possibility is that the mother fly can provide the eggs with specific bacteria. For example, there has been a study where it was shown that female houseflies could deposit an endosymbiotic bacterium on eggs in order to regulate further oviposition^51^. Another explanation could be that the mothers can locally deposit bacteria on the substrate where they lay their eggs, indirectly passing on members of their microbiota to their offspring. Simultaneous sampling from the mother, the eggs and the oviposition environment would help to test such a hypothesis and further investigate the ways in which houseflies keep certain bacteria through generations.

Moving on to the housefly-associated revealed fungal communities, we found a less diverse community composition of the fungal microbiotas. Two yeasts assigned to the taxa *Trichosporon* sp. and *Candida tropicalis* were detected in all larvae and pupae, and the substrate was provided to all housefly generations of all strains. Furthermore, these taxa were not detected in the samples of wild-caught housefly adults, whereas they were detected in the laboratory-reared adults. *Trichosporon* spp. is common basidiomycetous yeast-like fungi widely found in nature in a wide variety of habitats, whereas clinical isolates have been characterized as opportunistic pathogens causing superficial infections^52^. *C. tropicalis* is also a common fungus related to food spoilage, and some strains have been associated with invasive infections^53^. Both fungi were acquired through the diet, indicating that provided feed is pivotal for laboratory-reared flies. In general, the fungal housefly microbiota seems to be dependent on the provided diet, highlighting the importance of the rearing substrate to prevent any contamination risk.

### Environmental microbial transmission routes and the effect of microbiota disturbance on house fly health

Two main findings of this study, the environmental influence on the housefly gut microbiota and the consistency of the bacterial communities harbored in the gut in a given habitat, lead us to ask further questions on the underlying bacterial transmission routes. Rearing flies for multiple generations could help examine the long-term effect of various substrates on the acquired microbiota and investigate the extent to which the immediate environment shapes the gut bacterial communities. Furthermore, we should investigate how simple these communities can become without endangering the house fly health and fitness. We should also determine which symbionts are essential for adequate housefly development and how this depends on diet type. Also, disturbance of the housefly's natural microbiota, for example by introducing potentially pathogenic microbes into the larval environment, could help us look into how and to what extent the microbial gut community protects the housefly against pathogenic environmental microbes.

The recurrent bacterial communities within the same housefly strain and the possible interaction of bacterial and fungal communities could also mean that there are strong filtering mechanisms maintaining a significant set of commensal microbes in the gut. In the case of the housefly larvae, it has been shown that there is large capacity for microbial biomass bioconversion on organic waste streams without endangering fly health^54^. Moreover, rearing housefly larvae on poultry manure led to reduced substrate bacterial content and enhanced larval development^9^. It is yet to be studied if the housefly microbiome is resilient in environmental conditions, and the microbial community members could play a decisive role in rest stream remediation and supporting insect health in different habitats.

## Conclusions

The results of the present study indicate that *M. domestica* can associate with a wide range of microbes. The population sampled from its natural habitat continued to harbor a more diverse microbial community compared with two laboratory strains, even when reared in the laboratory for four consecutive generations, showing the influence of habitat on the internal housefly microbiota. Nevertheless, all strains shared some specific bacterial taxa, which have been characterized as housefly-associated and could be necessary for development. The fungal microbiota, on the other hand, was less diverse and the recurrent taxa found in the study were acquired by the houseflies through feeding, showing that the identified fungal taxa are probably transient members of the microbiota. Further research is needed to investigate what is the long-term effect of various substrates/environments on the housefly microbiota and what functions the acquired microbes might serve.

## Supplementary Information

Supplementary Information 1.

Supplementary Information 2.

Supplementary Information 3.

## Data Availability

All 16SrRNA gene and ITS1 sequencing data have been deposited to the Sequence Read Archive (SRA) under BioProject ID PRJNA806649.
