# Supplementary material for: Houseflies harbor less diverse microbiota under laboratory conditions but maintain a consistent set of host-associated bacteria
Source: Sci Rep. 2022 Jul 1;12:11132. doi: 10.1038/s41598-022-15186-7 (PMC9249849; doi:10.1038/s41598-022-15186-7)
Supplement: Supplementary file 1 — Supplementary Information 1. [file 41598_2022_15186_MOESM1_ESM.docx]

**Houseflies harbor less diverse microbiota under laboratory conditions but maintain a consistent set of host-associated bacteria**

Anna Voulgari-Kokota*, Leo W. Beukeboom, Bregje Wertheim, Joana Falcao Salles

Groningen Institute for Evolutionary Life Sciences (GELIFES), University of Groningen, P.O, Box 11103, 9700 CC, Groningen, the Netherlands

*corresponding author: a.voulgari.kokota@rug.nl

**Sampling scheme**

For every housefly strain and every generation, we kept three identical cages with adults (19×11.5×11.5 cm). Adults were provided with water, sugarwater and milk powder *ad libitum.* After obtaining eggs from every generation, we set up three cylindrical containers (11x11.5 cm) with eggs for every strain, keeping the same eggs/substrate ratio (4eggs/1g). Larvae, pupae and newly emerged adults were sampled from these containers simultaneously for all strains.

| Housefly strain | Number of generations reared in the laboratory before sampling | Geographic origin | Developmental stage | Replicates per generation |
| --- | --- | --- | --- | --- |
| GK0 | 0 | Gerkesklooster , Netherlands | Eggs (50mg) | 1 |
|  |  |  | Three-day old larvae | 3 |
|  |  |  | Two-day old pupae | 3 |
|  |  |  | Newly emerged adults | 3 |
|  |  |  | Three-day old adults | 3 |
| GK50 | >50 | Gerkesklooster, Netherlands | Eggs (50mg) | 1 |
|  |  |  | Three-day old larvae | 3 |
|  |  |  | Two-day old pupae | 3 |
|  |  |  | Newly emerged adults | 3 |
|  |  |  | Three-day old adults | 3 |
| SP100 | >100 | Barcelona, Spain | Eggs (50mg) | 1 |
|  |  |  | Three-day old larvae | 3 |
|  |  |  | Two-day old pupae | 3 |
|  |  |  | Newly emerged adults | 3 |
|  |  |  | Three-day old adults | 3 |
|  | | | Sample sum per generation | 39 |
|  | | | generations | x 4 |
|  | | | total number of samples | 156 |

**Table.** Housefly strains and developmental stages sampled
