# Supplementary material for: Houseflies harbor less diverse microbiota under laboratory conditions but maintain a consistent set of host-associated bacteria
Source: Sci Rep. 2022 Jul 1;12:11132. doi: 10.1038/s41598-022-15186-7 (PMC9249849; doi:10.1038/s41598-022-15186-7)
Supplement: Supplementary file 2 — Supplementary Information 2. [file 41598_2022_15186_MOESM2_ESM.docx]

**Houseflies harbor less diverse microbiota under laboratory conditions but maintain a consistent set of host-associated bacteria**

Anna Voulgari-Kokota*, Leo W. Beukeboom, Bregje Wertheim, Joana Falcao Salles

Groningen Institute for Evolutionary Life Sciences (GELIFES), University of Groningen, P.O, Box 11103, 9700 CC, Groningen, the Netherlands

*corresponding author: a.voulgari.kokota@rug.nl

The sequence alignment and the phylogenetic reconstructions were performed using the function "build" of ETE3 v3.1.1 (Huerta-Cepas *et al*., 2016) as implemented on the GenomeNet (<https://www.genome.jp/tools/ete/>). The alignment of the partial cytochrome oxidase subunit I (COI) gene sequences (626 bp) was performed with MAFFT v6.861b with the default options (Katoh and Standley, 2013). The maximum likelihood tree was inferred using PhyML v20160115 ran with model GTR and parameters: --alpha e -o tlr -f m --bootstrap 100 --nclasses 4 --pinv e (Guindon *et al*., 2010). Branch supports are computed out of 100 bootstrapped trees.

midpoint rooted tree:


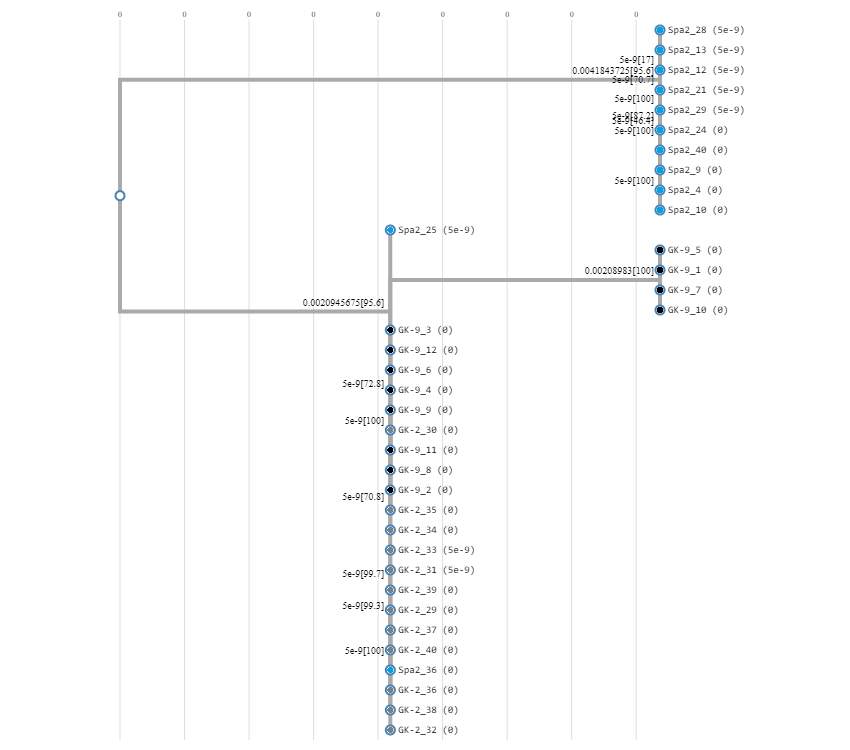

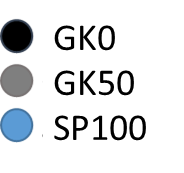


Huerta-Cepas, J., Serra, F. & Bork, PETE 3: Reconstruction, Analysis, and Visualization of Phylogenomic *Data. Mol. Biol. Evol.* **33**(6), 1635–1638 (2016).

Katoh, K. & Standley, D. M. MAFFT multiple sequence alignment software version 7: improvements in performance and usability. *Mol. Biol. Evol.* **30**(4), 772–780 (2013).

Guindon, S., Dufayard, J. F., Lefort, V., Anisimova, M., Hordijk, W. & Gascuel, O. New algorithms and methods to estimate maximum-likelihood phylogenies: assessing the performance of PhyML 3.0. *System. Biol.* **59**(3), 307–321 (2010).
