# Supplementary material for: Houseflies harbor less diverse microbiota under laboratory conditions but maintain a consistent set of host-associated bacteria
Source: Sci Rep. 2022 Jul 1;12:11132. doi: 10.1038/s41598-022-15186-7 (PMC9249849; doi:10.1038/s41598-022-15186-7)
Supplement: Supplementary file 3 — Supplementary Information 3. [file 41598_2022_15186_MOESM3_ESM.docx]

**Houseflies harbor less diverse microbiota under laboratory conditions but maintain a consistent set of host-associated bacteria**

Anna Voulgari-Kokota*, Leo W. Beukeboom, Bregje Wertheim, Joana Falcao Salles

Groningen Institute for Evolutionary Life Sciences (GELIFES), University of Groningen, P.O, Box 11103, 9700 CC, Groningen, the Netherlands

*corresponding author: a.voulgari.kokota@rug.nl

**Supplementary material**

**qPCR data**

**
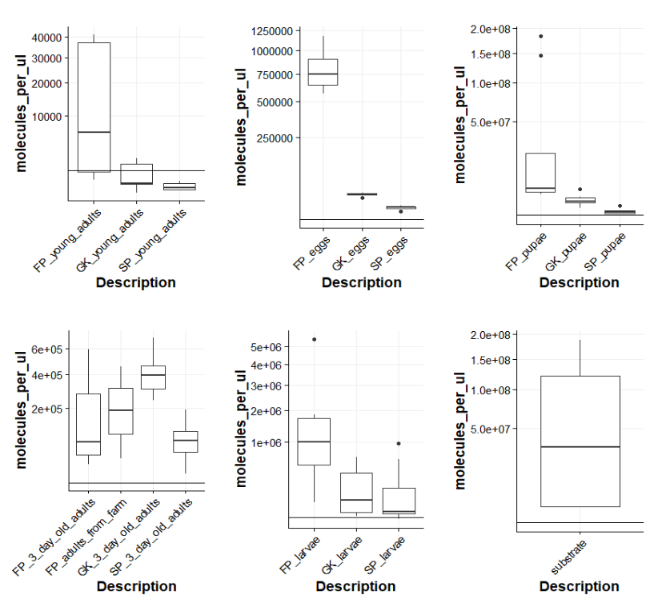
**

Amplification traces for the bacterial 16SrRNA gene.The horizontal line represents the copy number lower limit (1000).


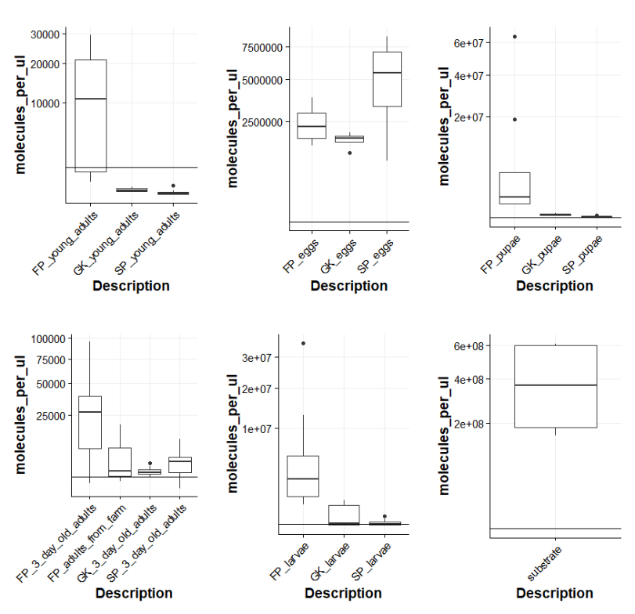


Amplification traces for the fungal ITSrRNA gene. The horizontal line represents the copy number lower limit (1000).

**Sequencing rarefaction curves**


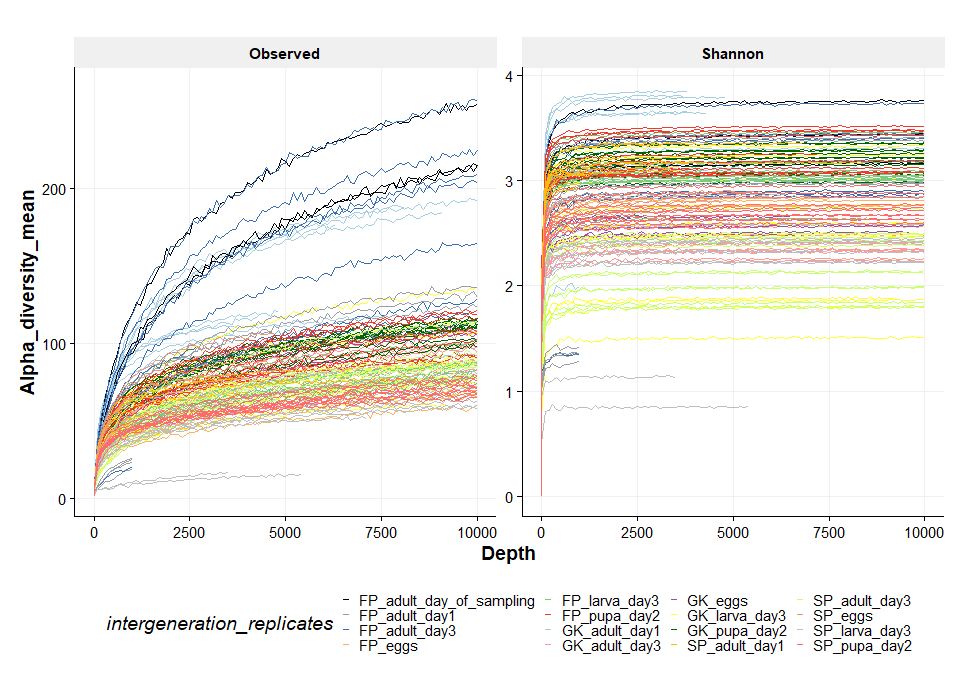


Alpha diversity plotted against sequencing depth represented by number of reads for the bacterial 16SrRNA.


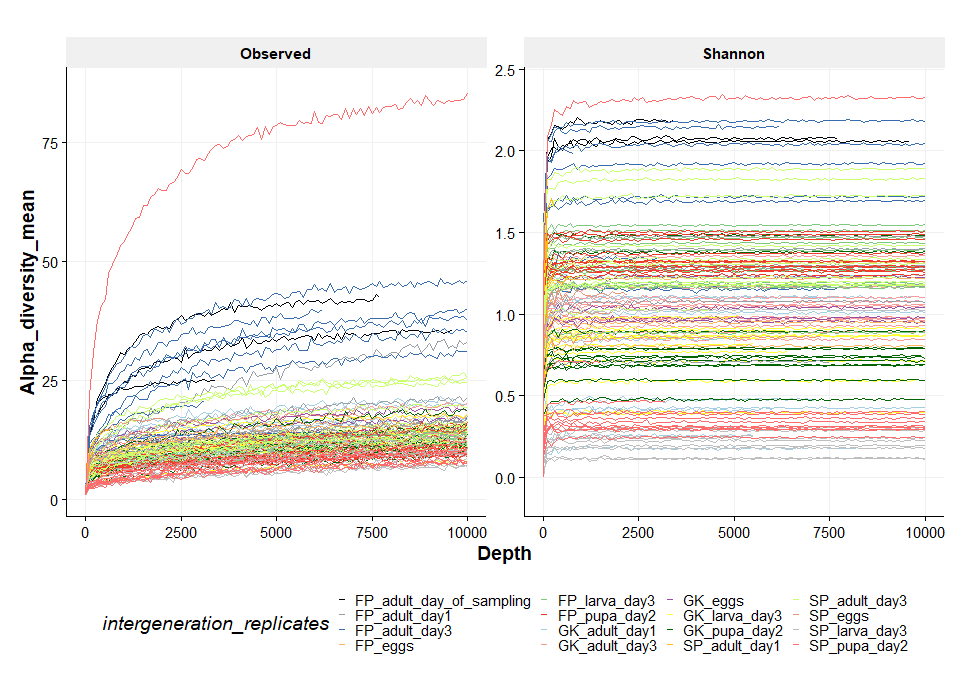


Alpha diversity plotted against sequencing depth represented by number of reads for the fungal ITSrRNA.

**
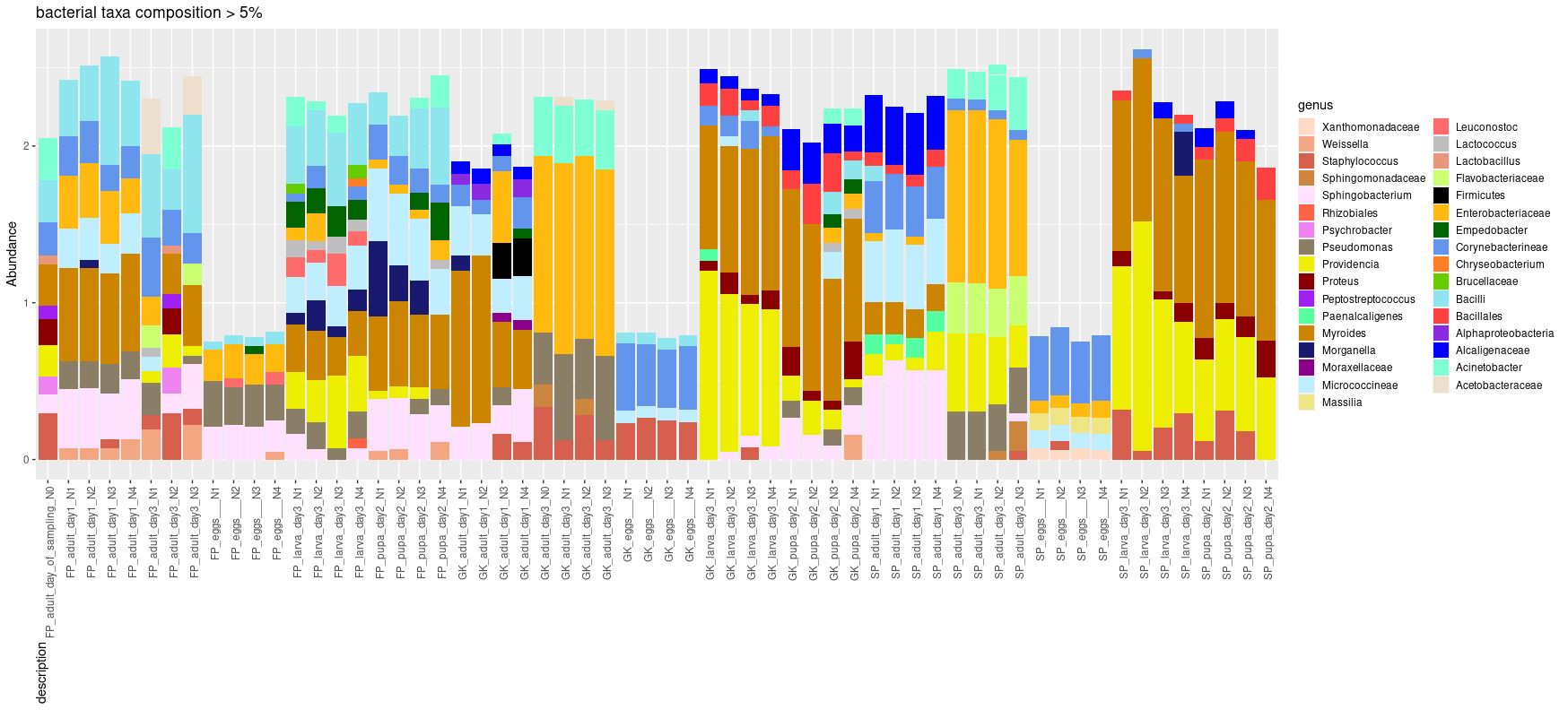

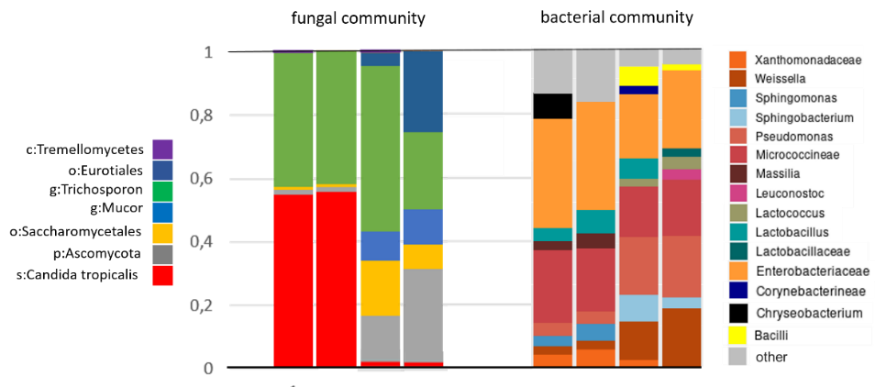
Microbial composition**

a. Microbial community of the provided substrate

**
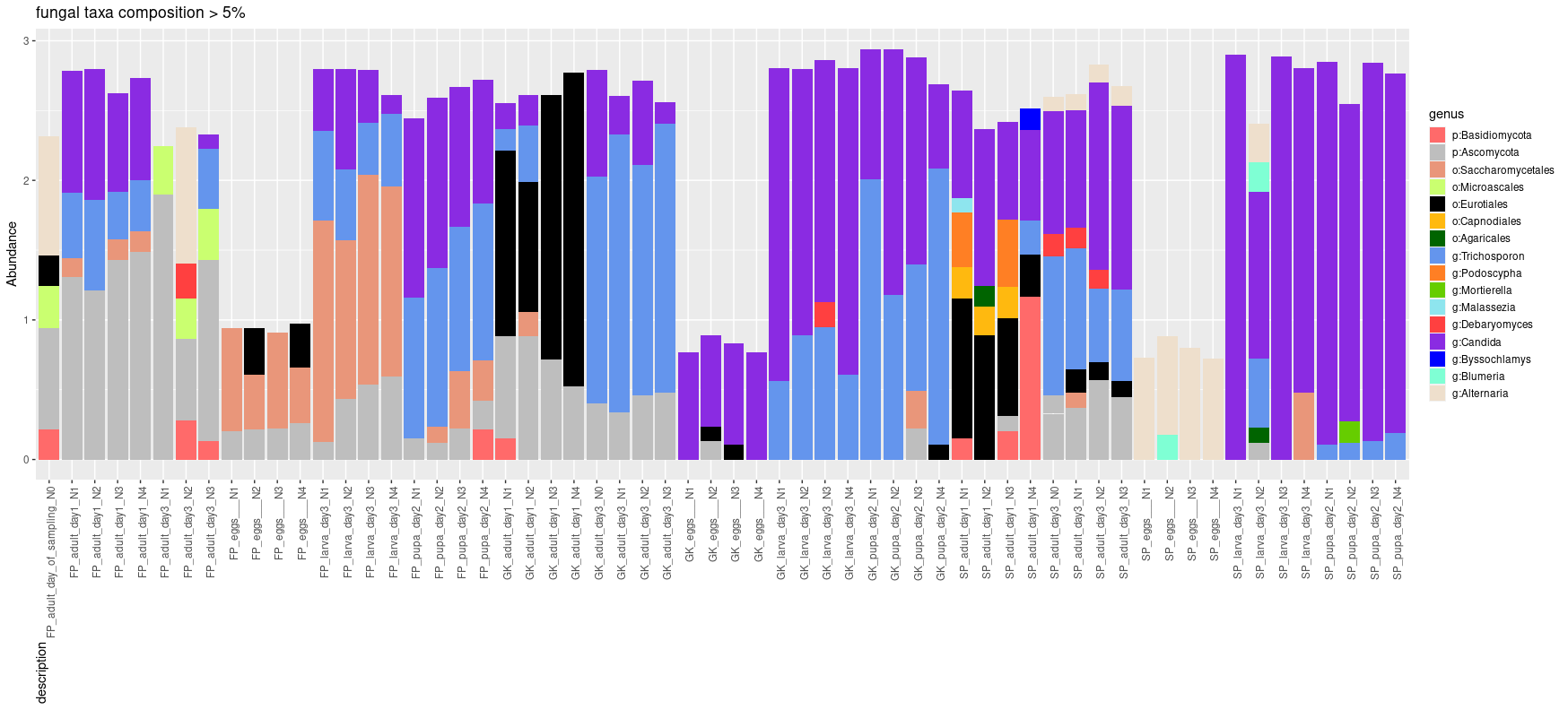
** b. Bacterial community composition of all housefly samples

c. Fungal community composition of all housefly samples

a) Microbial composition of the egg-laying substrate provided to each from the four generations of all housefly strains. Each bar stands for the batch of fresh substrate for each generation of flies. b) Bacterial community composition of all housefly samples and c) fungal community composition of all housefly samples. Each bar stands for all replicates of the same housefly strain x developmental stage x generation samples. Bacterial and fungal taxa are included in the barplots, if their relative abundance is detected in more than 5% in the whole dataset.
